# Co-W Barrier Layers for Metallization of Copper Interconnects: Thermal Performance Analysis

**DOI:** 10.3390/nano12101752

**Published:** 2022-05-20

**Authors:** Bruno M. C. Oliveira, Ruben F. Santos, Ana P. Piedade, Paulo J. Ferreira, Manuel F. Vieira

**Affiliations:** 1 Department of Metallurgical and Materials Engineering, University of Porto, Rua Dr. Roberto Frias, 4200-465 Porto, Portugal; rbns@fe.up.pt (R.F.S.); mvieira@fe.up.pt (M.F.V.); 2 LAETA/INEGI—Institute of Science and Innovation in Mechanical and Industrial Engineering, Rua Dr. Roberto Frias, 4200-465 Porto, Portugal; 3 CEMMPRE—Department of Mechanical Engineering, University of Coimbra, 3030-788 Coimbra, Portugal; ana.piedade@dem.uc.pt; 4 International Iberian Nanotechnology Laboratory, Av. Mestre José Veiga, 4715-330 Braga, Portugal; paulo.ferreira@inl.int; 5 Materials Science and Engineering Program, University of Texas at Austin, Austin, TX 78712, USA; 6 Mechanical Engineering Department and IDMEC, Instituto Superior Técnico, University of Lisbon, Av. Rovisco Pais, 1049-001 Lisboa, Portugal

**Keywords:** barrier layers, dewetting, cobalt tungsten, copper metallization

## Abstract

The back-end-of-line (BEOL) copper interconnect structure has been subjected to downscaling for the last two decades, while the materials used for conforming and assuring its physical integrity during processing have faced significant obstacles as the single-digit nanometer process node is implemented. In particular, the diffusion barrier layer system comprised of Ta/TaN has faced major constraints when it comes to the electrical performance of the smaller Cu lines, and thus alternative formulations have been investigated in recent years, such as Ru-Ta or Co-W alloys. In this work, we assess how PVD (physical vapor deposition) deposited equimolar Co-W films perform when exposed to different vacuum annealing temperatures and how these films compare with the Ta adhesion layer used for Cu seeding in terms of dewetting resistance. The stacks were characterized using scanning electron microscopy (SEM), X-ray diffraction (XRD), transmission electron microscopy (TEM) and scanning transmission electron microscopy (STEM) coupled with energy dispersive X-ray spectroscopy (EDX) mapping. The Cu film at the surface of the Cu/Co-W system exhibited grain growth starting at 300 °C, with the formation of abnormally large Cu grains starting at 450 °C. Sheet resistance reached a minimum value of 7.07 × 10^−6^ Ω/sq for the Cu/Co-W stack and 6.03 × 10^−6^ Ω/sq for the Cu/Ta stack, both for the samples annealed at 450 °C.

## 1. Introduction

The replacement of aluminum by copper as the metal of choice for ULSI (ultra large scale integration) interconnects in the late 1990s has led to a significant increase in the research of copper films, particularly with respect to downscaling. Currently, as the miniaturization of circuits is capable of processing nodes below 10 nm, the limitations inherent to the production of these structures has further fostered the need for new materials to optimize the performance and manufacturability of copper interconnects [1,2,3]. In this regard, the use of Ta/TaN adhesion/barrier layer systems is becoming a structural limitation [4]. In fact, the use of Ta/TaN restricts the minimum Cu seed layer thickness due to copper overhang after electroplating, which is not compatible with the continuous miniaturization process in the nanoelectronics industry [5]. The high interfacial energy of the Ta/Cu interface also limits the performance of systems based on this adhesion layer due to the electromigration resistance limit of the interconnect wires produced using these materials [6]. A variety of alternative materials to these Ta/TaN films used in copper metallization have been proposed, such as Co, Ti-W, Co-W, or Ru based systems like Ru-Ta or Ru-W [6,7,8,9,10,11,12].

The use of Co-W as a candidate system for replacing the Ta/TaN has been suggested for seedless Cu metallization, potentially suppressing the need for a copper seed layer deposited by PVD (physical vapor deposition), thus eliminating one of the manufacturing steps necessary for the production of each copper interconnect layer [7,13]. While the work of Su et al. [7] showed the use of Co-W as a directly plateable material for diffusion barrier layers, the effect of the annealing treatment on the copper layer deposited over the Co-W barrier was not addressed in detail. The authors showed the effects of a rapid thermal annealing (RTA) treatment of Cu/Co-W films deposited over differently formulated Co-W alloys at 400 °C for 30 min, but an in-depth microstructural study of the substrates or films after annealing was not shown. Knowledge of the relation between the annealing conditions and its effects on the materials involved is crucial in assessing the viability of this solution as a new diffusion barrier material. 

In order to be a viable alternative for the deployment of new diffusion barrier liners, the Co-W layer should exhibit an interfacial energy with the underneath Cu film similar to the Ta/Cu interface, so that dewetting of the copper film does not occur during the annealing step, which is critical for the manufacture and operation of the devices [7]. It is also important to ascertain the electrical performance of the barrier layer and copper film stack, specifically pertaining to its overall electrical resistivity, as it provides an insight into the energy efficiency of the system and its capability to deal with the electrical current transport during service conditions. The fact that neither Co nor W should form compounds with Cu makes them an interesting alternative material for diffusion barrier layers. There is a reduced risk of creating phases that are not as electrically conductive as the metallic Cu film without a considerable loss to the adhesion strength of the interface [14].

In this work, we explore the evolution of the surface of copper films deposited over a Co-W barrier layer as a function of annealing temperature, as well as assess the effect of processing conditions on the electrical resistivity of the films. 

## 2. Materials and Methods

### 2.1. PVD Film Deposition

The metallic layers were deposited over a plasma-enhanced chemical vapor deposition (PECVD) (SPTS Technologies Ltd., Newport, UK) prepared 100 nm SiO_2_ surface of a 15 mm × 15 mm p-type boron doped single crystal Si (100) wafer piece (Silicon Valley Microelectronics, Santa Clara, CA, USA). The 25 nm thick Co-W films were deposited by co-sputtering Co and W targets (99.95%, Testbourne Ltd., Basingstoke, UK) for 600 s in a DC magnetron sputtering system (Kenosistec, Binasco, Italy), at 40 W power for each of the targets used, using a constant Ar flux of 20 sccm. These conditions aimed to produce a film with a 1:1 atomic ratio, as used in [13]. The Ta film representing the reference adhesion layer material was deposited at 100 W for 380 s to produce a film of equal thickness, with a base pressure of 6.4 × 10^−5^ Pa and working pressure of 6.9 × 10^−3^ Pa. Both layers were covered by a Cu layer deposited at 40 watts for 900 s, with a base pressure of 6.9 × 10^−3^ Pa.

### 2.2. Vacuum Annealing

The dewetting behavior of the films was analyzed by vacuum annealing treatments carried out inside an alumina tube furnace for one hour after reaching the set temperature, using a 5 °C/minute heating/cooling rate. Starting pressure of the annealing treatments was 1.20 × 10^−3^ Pa. The films’ surface was studied after treatments at 300, 450, 500, 550, 600, 650, and 700 °C.

### 2.3. Film Characterization

The surface structure observation was carried out using scanning electron microscopy (SEM, Thermo Fischer Scientific Quanta 400FEG ESEM and Thermo Fischer Scientific Quanta 650FEG ESEM, Waltham, MA, USA). The film surface evolution as a function of the annealing temperatures was evaluated by the grazing incidence X-ray diffraction (GIXRD) using Cu Kα radiation (λ = 0.15406 nm) at a 1.5° angle with a 0.04°·s^−1^ step. The interfacial region of the barrier layer/copper film was observed cross-section in a lamella prepared by a focused ion-beam (FIB, Thermo Fisher Helios 450S). The Cu film surface of these lamellae was preserved by using protective Pt layers deposited with electron and Ga^+^ ion beams prior to milling. These samples were characterized using aberration-corrected high angle annular dark-field scanning transmission electron microscopy (HAADF STEM, Thermo Fischer Scientific Titan G2 ChemiSTEM, at 200 kV) in conjunction with energy dispersive X-ray element mapping. Topography mapping of the Cu/Ta films was undertaken using atomic force microscopy (AFM, Veeco Metrology Multimode, Veeco Instruments Inc., Plainview, NY, USA) to evaluate the state of the film surface after annealing at high temperatures. 

The electrical resistivity of the films was determined using a four-point probe measurement system in a Van der Pauw configuration (Hall-Effect Measurement System Ecopia 5000, Ecopia, Gyeonggi-do, South Korea) at room temperature. Each sample was measured a total of ten times before being rotated 90°, after which a second set of ten additional measurements was carried out, with the final result being the average value of the total measurements of both orientations per sample.

## 3. Results and Discussion

### 3.1. Scanning Electron Microscopy

**Figure 1:**
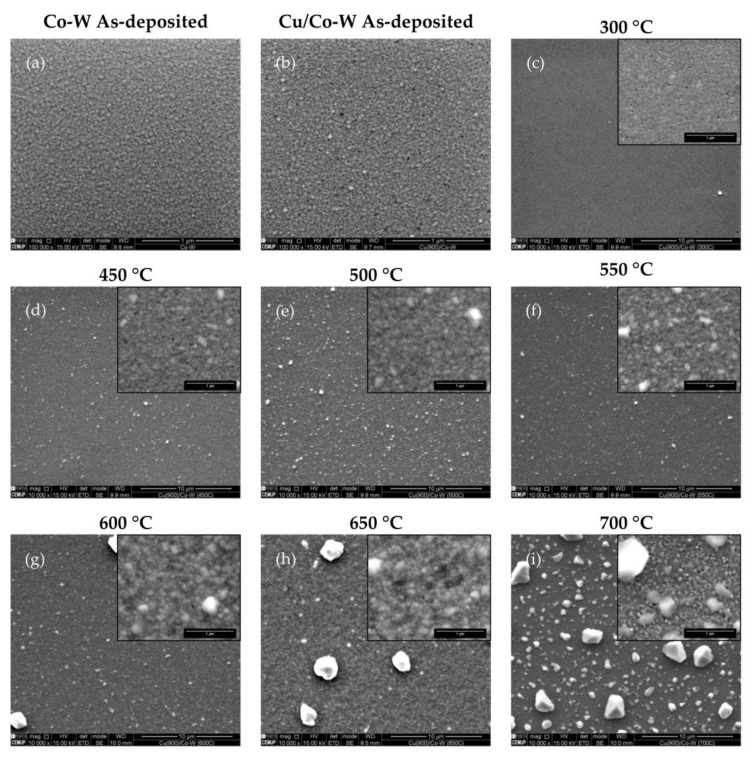
SEM secondary electron (SE) images of the Co-W (**a**) and Cu/Co-W film surfaces in the as-deposited state (**b**), and after annealing at 300 (**c**), 450 (**d**), 500 (**e**), 550 (**f**), 600 (**g**), 650 (**h**), and 700 °C (**i**). The insets show higher magnification images of the respective surface. Scale bar corresponds to 1 μm in images (**a**,**b**), and all higher magnification insets, while corresponding to 10 μm in (**c**–**i**) main images.

Figure 1a shows the surface of the Co-W layer in the as-deposited condition, showing a compact film throughout. The surface of all Cu films deposited over the Co-W samples, visible in Figure 1b–i, exhibited some pinholes of relatively uniform dimensions on the surface of the copper layer, even on the as-deposited condition. This surface irregularity could indicate contamination of the deposition chamber during coating or merely be the result of the reproduction of surface defects present at the original substrate surface over which the sputtering process was undertaken [15,16,17]. The absence of this type of defect in the as-deposited Co-W layer in Figure 1a indicates that pinhole formation for the deposition conditions employed was likely associated with the growth of the Cu film, potentially due to impurity contamination of the Cu target used or due to epitaxial growth of the Cu film from the Co-W surface. As the temperature of the isothermal annealing stage rises, these round holes dispersed throughout the Cu surface did not exhibit discernible growth, suggesting that they were not associated with mechanisms for residual stress relaxation or any other thermal-activated mechanisms that affected atomic mobility during annealing. The copper film deposited over the Co-W barrier layer exhibited grain growth after annealing at 300 °C (Figure 1c). As the annealing temperature increased up to 600 °C, abnormally large grains with irregular shapes started emerging from the relatively smooth surface of the film, whilst still maintaining full substrate coverage, as shown in Figure 1d–f). It is possible to notice that the size of these irregularly-shaped grains increased with annealing temperature due to the corresponding increase in atomic mobility on the copper film.

**Figure 2:**
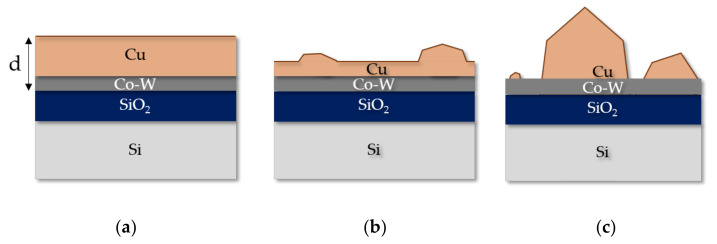
Cross-section representation of the Cu/Co-W stack in the as-deposited condition (**a**), after initial dewetting of the Cu layer at the surface, following annealing (**b**) and after complete dewetting of the Cu film (**c**).

**Figure 3:**
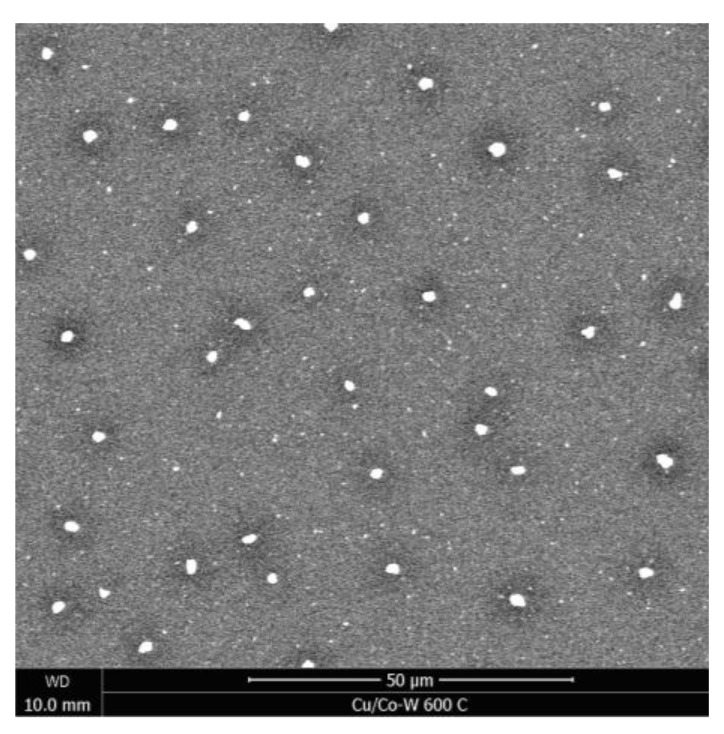
SEM BSE image of the Cu/Co-W film after 600 °C vacuum annealing. The darker regions around coarse Cu grains highlight the localized thinning of the film.

In Figure 1h, some areas of the substrate underneath the Cu film are exposed, with Figure 1i exhibiting complete dewetting of the copper film, as the copper atoms previously covering the surface of the film have been consumed to foster the growth of larger grains, as represented in Figure 2, where d corresponds to the combined thickness of the Cu film and the diffusion barrier layer. In Figure 3, the backscattered electron (BSE) signal highlights how the neighboring regions of the protruding Cu grains have a lower atomic number, reflecting the localized Cu atom depletion.

**Figure 4:**
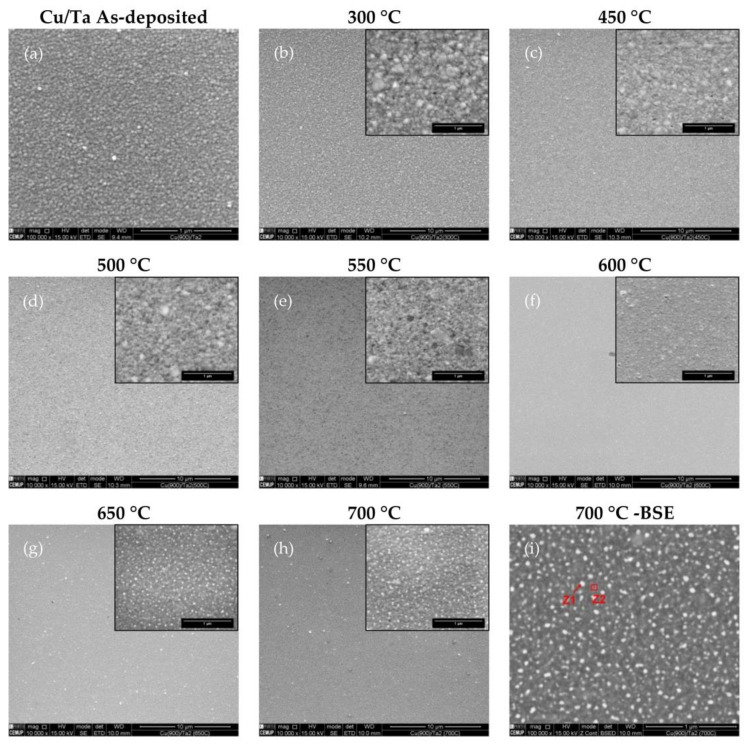
SEM SE of the Cu/Ta film surfaces in the as-deposited state (**a**) and after annealing at 300 (**b**), 450 (**c**), 500 (**d**), 550 (**e**), 600 (**f**), 650 (**g**), and 700 °C (**h**). The insets show higher magnification images of each surface. SEM BSE Cu/Ta surface of the films after annealing at 700 °C, with indication of areas scanned for the EDS spectral analysis (**i**). Scale bar corresponds to 1 μm in image (**a**) and all higher magnification insets, while corresponding to 10 μm in (**b**–**i**) main images.

**Figure 5:**
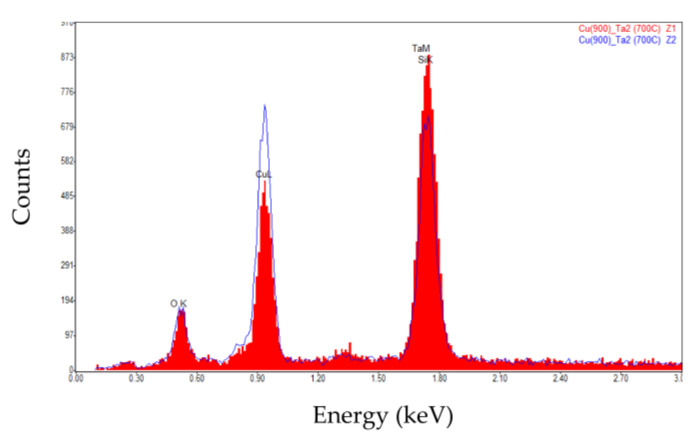
The EDS spectra of the two distinct zones marked in Figure 4i of the surface of the Cu/Ta film after annealing at 700 °C showing lower Cu content on the brighter constituent.

In Figure 4, it is possible to notice how the annealing temperature affects the surface evolution of the Cu films deposited over the Ta layer. From room temperature up until 500 °C, seen in Figure 4a–d, there are no appreciable changes to the Cu film surface. Above this temperature, it is possible to see a change in contrast at the surface, Figure 4e,f, which hints at a possible change in chemical composition, with the possibility of Ta diffusion towards the Cu film surface, as shown in [18]. Above the 650 °C annealing temperature, it is possible to verify that the chemical composition of the Cu film surface has been altered, as evidenced by the appearance of white dotted regions dispersed throughout the observed surface (Figure 4g,h). The appearance of these dotted regions at this temperature range is likely linked to the Ta diffusion towards the surface of the Cu film, identified in [19] to happen as early as 630 °C. EDS analysis results of both type of constituents, shown in Figure 5, indicates that the brighter regions have a Cu deficit when compared to the darker matrix over which they are dispersed, consistent with the presence of a higher atomic weight material such as Ta, which has seemingly diffused to the surface. No analyzed temperature conditions have apparently initiated Cu film dewetting for this system. 

**Figure 6:**
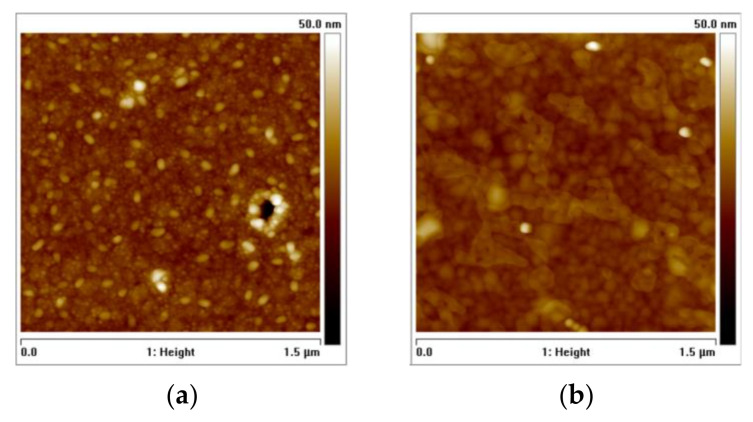
AFM topographic images for the Cu/Ta stack annealed at 600 °C (**a**) and at 650 °C (**b**).

The formation of lighter particles above the 600 °C mark for the Cu/Ta stack warranted further investigation using AFM, as seen in Figure 6. This result can be associated with the respective SEM EDS analysis, thus demonstrating that the integrity of the copper film was seemingly compromised by Ta diffusion from the bottom layer up to the surface.

### 3.2. X-ray Diffraction

**Figure 7:**
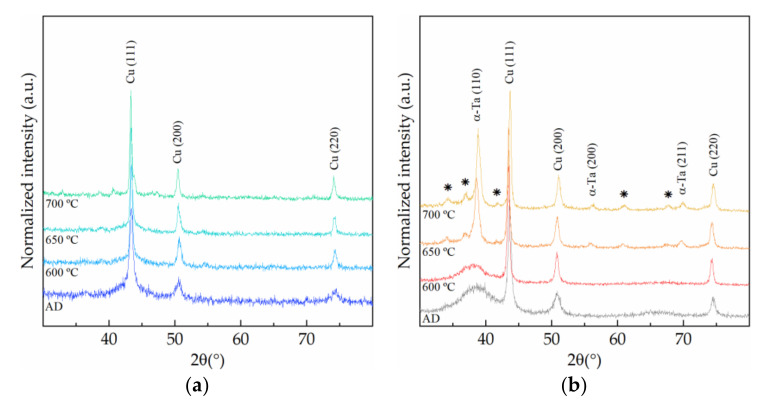
X-ray diffractograms of the Cu/Co-W (**a**) and the Cu/Ta (**b**) stacks in different annealing conditions.

The diffractograms of the Cu/Co-W stacks in Figure 7a show a slight sharpening and increase in intensity of the two first peaks at 43.40° and 50.52°, consistent with the Cu (111) and (200) orientations, with annealing temperature, which can be attributed to an increase in the grain size of the Cu film on the top of the stack. The absence of any signal specific to Co or W suggests that the barrier layer did not diffuse towards the top of the Cu film. The Cu/Ta stack, by contrast, shows the formation of three new peaks at 34.08°, 36.85°, and 38.56° for annealing temperatures above 600 °C, in addition to the characteristic Cu peaks mentioned earlier. This is compatible with the diffusion of Ta from the interface to the surface of the Cu film, as initially hypothesized by the corresponding SEM image analysis. Furthermore, in [20], the authors identified TaSi_2_ as the first phase forming at Cu/Ta interfaces after annealing at 600 °C, with the formation of other Cu and Ta silicides also being compatible with the new peaks highlighted in Figure 7b [21].

### 3.3. Transmission Electron Microscopy

**Figure 8:**
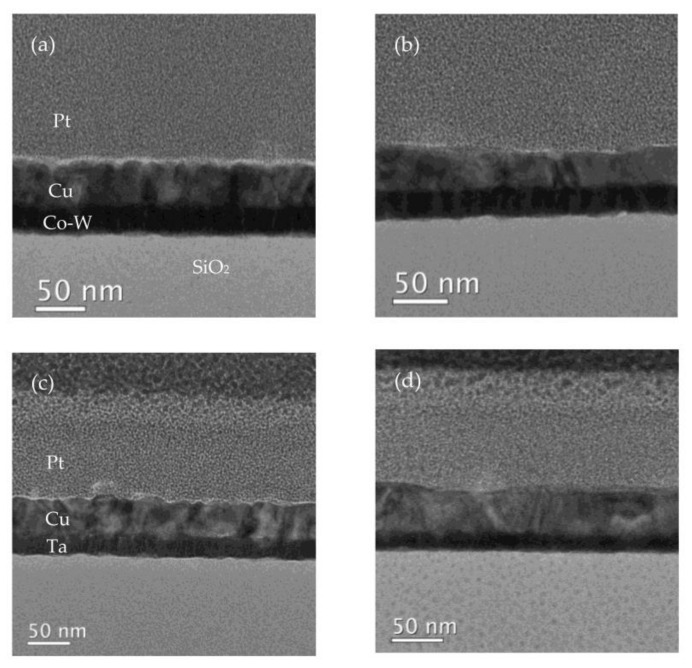
BF TEM images of the Cu/Co-W layers in the as-deposited (**a**), after annealing at 600 °C (**b**), and of the Cu/Ta system in the as-deposited (**c**) and after annealing at 600 °C (**d**).

In Figure 8, the results of bright-field (BF) TEM of the FIB milled lamellae are shown. It is possible to notice how the vacuum annealing treatments affected the Cu film surface, with the annealed samples showing larger Cu grains. 

**Figure 9:**
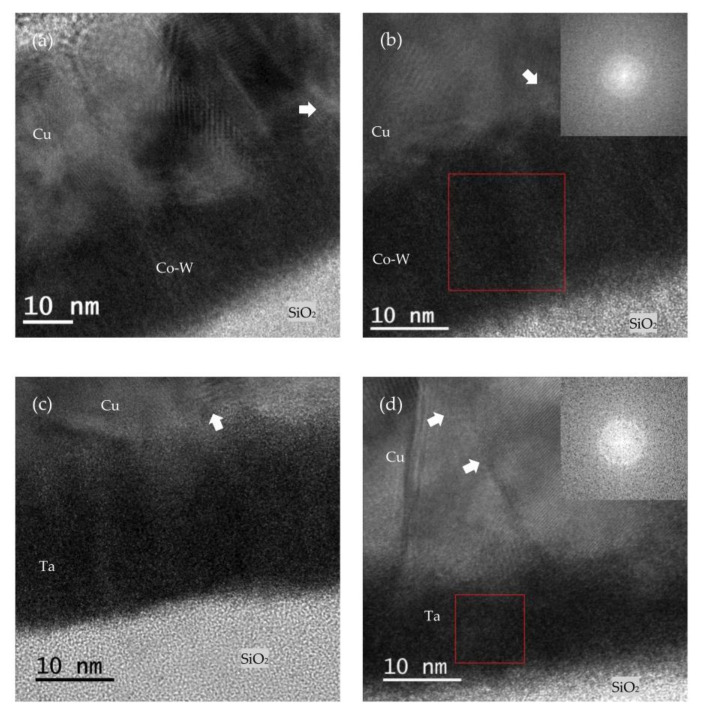
HR BF TEM images of the Cu/Co-W (**a**) and Cu/Ta (**c**) in the as-deposited, and after annealing at 600 °C, with FFT of the highlighted area on the inset, for the Cu/Co-W (**b**) and Cu/Ta (**d**) systems. Arrows indicate the location where lattice fringes can be seen.

The higher magnification images visible in Figure 9 show that while the Cu layers exhibited crystallinity in some regions, as evidenced by the highlighted lattice fringes, the substrate Co-W and Ta layers remained mostly amorphous after annealing at 600 °C, as confirmed by the fast Fourier transform (FFT).

**Figure 10:**
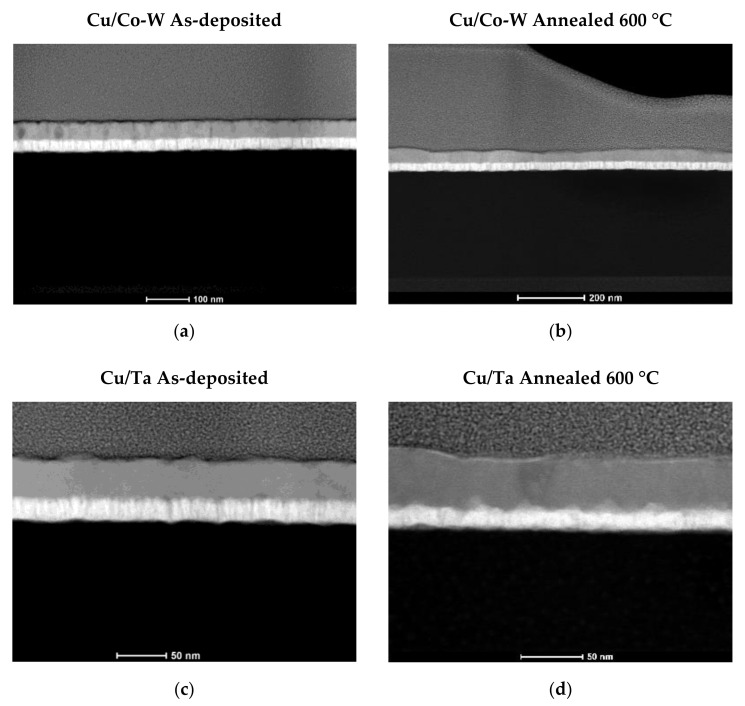
HAADF STEM images of the Cu/Co-W sample in the as-deposited state (**a**), after annealing at 600 °C (**b**), as well as of the as-deposited Cu/Ta (**c**) and after 600 °C vacuum annealing (**d**).

Improved contrast imaging for the measurement of the average film thickness of all films was achieved using HAADF STEM images, partially shown in Figure 10. In Figure 10a,c, it is possible to see a Cu surface with small thickness variation throughout the observable length, but the images visible in Figure 10b,d and corresponding to the annealed samples, show variation in Cu film thickness.

It is important to note that the annealed Cu/Ta interface lacks as an accurate definition of the contours of the films as the other observed conditions, possibly reflecting the occurrence of some Ta diffusion at the respective temperature of 600 °C, as suggested by the SEM BSE imaging shown earlier.

Both substrate films exhibit a rather columnar amorphous structure in the as-deposited condition, which was reduced by the annealing treatments, as seen in Figure 10. Changes to mean Cu film thickness in both systems are attributed to different main reasons, namely lateral Cu diffusion to sustain the growing grains deposited over the Cu/Co-W film, as explained by Thompson in [22], and a loss in contrast at the upper edges of the Ta film, associated with Ta diffusion toward the Cu film above, for the Cu/Ta system. The thicknesses of the various films displayed in Figure 10 are shown in Table 1. Analysis of the variation in Cu film thickness for both substrate materials further reinforces the higher dewetting tendency of the Co-W system when compared to the Ta adhesion layer.

**Figure 11:**
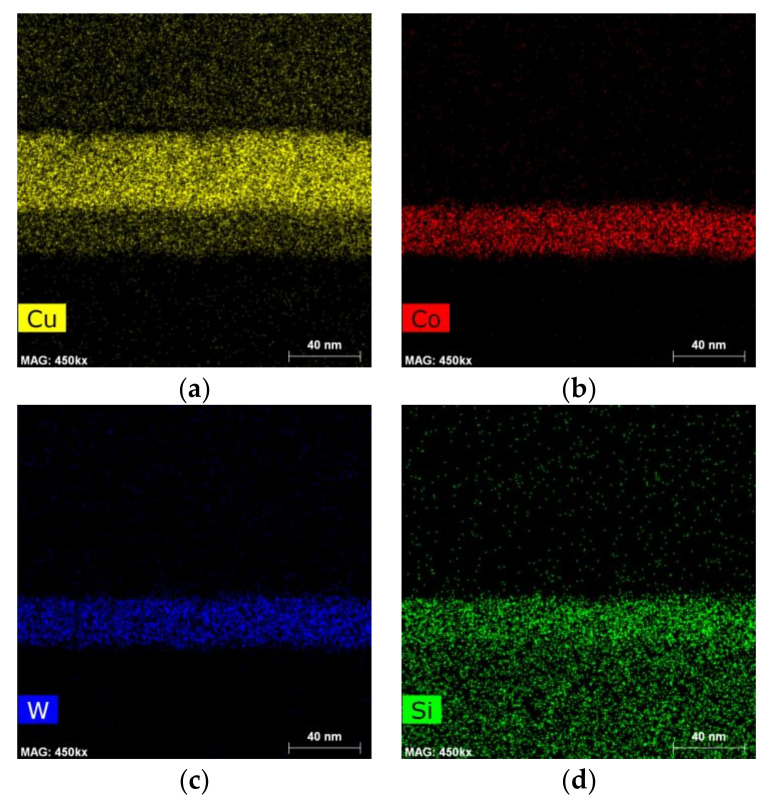
EDX mapping of Cu (**a**), Co (**b**), W (**c**), and Si (**d**) for the as-deposited Cu/Co-W system.

**Figure 12:**
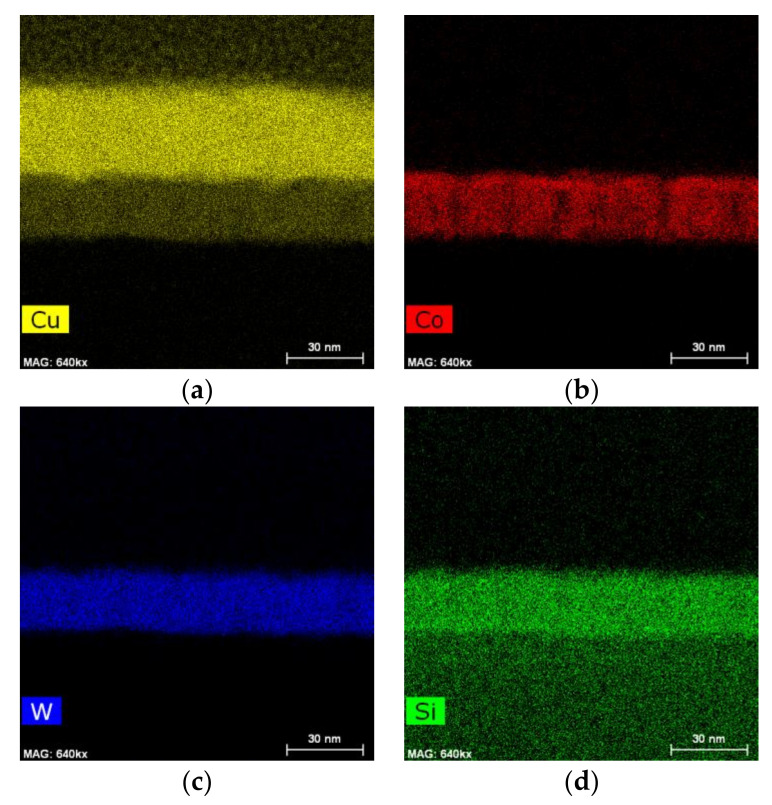
EDX mapping of Cu (**a**), Co (**b**), W (**c**) and Si (**d**) for the Cu/Co-W system after annealing at 600 °C.

The EDX mapping of the Cu/Co-W system in Figure 11 and Figure 12 shows that the barrier layer was effective at inhibiting diffusion from any of the elements present to upper or lower layers, with no discernible formation of any compounds at any of the visible interfaces. The Cu regions visible in Figure 11a and Figure 12a appear constricted to their original locations, as do the Co and W maps visible in Figure 11b, Figure 12b and Figure 11c, Figure 12c, respectively. However, the top of the barrier layer seems slightly enriched in Co after annealing, as shown in Figure 12b. This is possibly the result of a change in Co miscibility of the film for this atomic ratio at the temperature range studied. The apparent presence of Si atoms at the barrier layer in Figure 11d and Figure 12d is attributed to the overlap of the W M peak with the Si K peak at 1.774 and 1.739 keV, respectively, even though the W signal was collected using only the L α energy at 8.396 keV.

**Figure 13:**
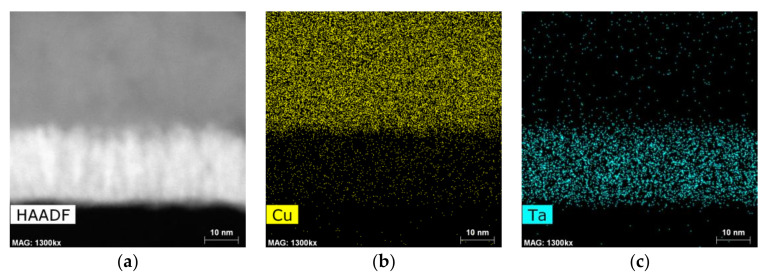
HAADF image of the Cu/Ta sample in the as-deposited state (**a**); EDX mapping of Cu (**b**), and Ta (**c**) for the corresponding observable area.

The EDX mapping of the as-deposited Cu/Ta stack seen in Figure 13 shows a well-delineated interface between the Cu and the Ta signals.

**Figure 14:**
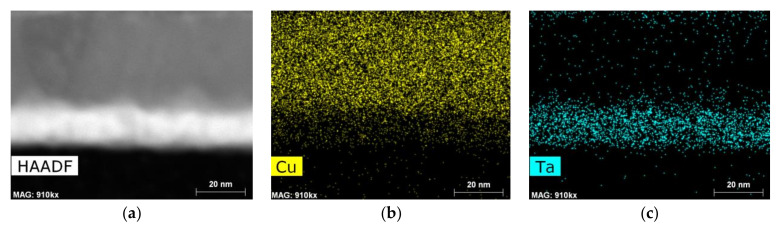
HAADF image of the Cu/Ta sample after vacuum annealing at 600 °C (**a**); EDX mapping of Cu (**b**) and Ta (**c**) for the corresponding observable area.

The 600 °C annealing temperature proved high enough to affect the original structure of the Cu/Ta interface, as indicated by the loss in atomic number contrast at the interface of the Ta and Cu films visible in Figure 14a. The Cu film did not seem to diffuse towards the substrate, as seen in Figure 14b. However, the Cu/Ta interfacial evaluation of the annealed films exhibited a residual infiltration of Ta onto the bottom of the Cu film, as seen on Figure 14c. This suggests the early stages of Ta diffusion, as reinforced by the serrated geometry the interface has developed, as seen in the HAADF image.

### 3.4. Electrical Characterization

**Figure 15:**
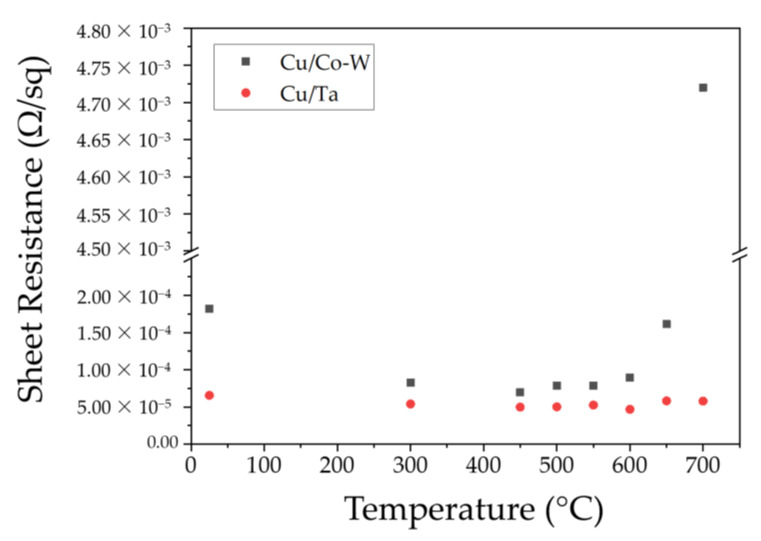
The sheet resistance of the Cu/Co-W and Cu/Ta stacks as a function of vacuum annealing temperature.

As interconnects are downscaled, the contribution of the barrier layer system to the overall resistivity also increases. A low electrical resistivity is of paramount importance for a barrier layer material, particularly when dealing with films of very small thicknesses, since the overall resistivity of the barrier layer/liner-interconnect system is adversely affected by a decrease in total thickness, due to increases in diffuse electron scattering [23,24,25]. The results of electrical sheet resistance measurements are shown in Figure 15.

The sheet resistance of both materials decreases for the first two temperatures was evaluated as the copper films gradually undergo grain growth and diffuse electron scattering decreases as a consequence of a reduction in total grain boundary scattering. The minimum sheet resistance measured for the Cu/Co-W system was 7.07 × 10^−6^ Ω/sq, measured at the system annealed at 450 °C. Above 450 °C, the Cu/Co-W system has an increase in resistance as the overall Cu film thickness decreases and diffuse electron surface scattering becomes more pronounced. Sheet resistance rises as dewetting of the copper layer progresses for higher annealing temperatures, as shown in Figure 15 above 550 °C. Meanwhile, the growth of the existing Cu grains is increasingly hampered by the decrease in film thickness due to the progress of dewetting. From this stage onwards, the total amount of Cu grains capable of carrying the electron current is decreasing and becoming farther apart, thus inhibiting efficient electron current flow along the surface of the film.

The Cu/Ta system displayed superior performance across all investigated temperature ranges, with its best resistance value of 6.03 × 10^−6^ Ω/sq found after annealing at 450 °C, with all remaining sheet resistance values measured not exhibiting considerable changes in behavior. It is important to note that the absence of the less electrically conductive barrier layer of TaN in the adhesion layer used in this work contributes to the overall system performance, which is not an accurate representation of the systems used in practical applications.

The *d* parameter used for resistivity calculation, representing the total metallic film thickness, was taken as the sum of the average values in Table 1 for both Cu and barrier films in each condition. Calculation of the electrical resistivity of each sample was achieved by multiplying the respective sheet resistance with said thickness value, with the results visible in Table 2.

Considering that TaN can exhibit resistivity values of 0.3 Ω∙cm at 2 nm thickness [26] or the 1.5689 × 10^−3^ Ω∙cm for a 97.64 nm thick film [27], it is worth noting that a Cu/TaN system is bound to exhibit higher resistivity values at similar thicknesses. In this way, while this Cu/Ta system displayed lower values than the Cu/Co-W alternative, a conventional dual-layer system comprised of a Ta adhesion layer with a TaN barrier layer should yield a higher sheet resistivity than that observed for the Co-W system at equivalent thickness due to the aforementioned TaN electrical properties. In this way, Co-W shows the potential to become a viable material for integration as a substrate for Cu interconnects. The presence of a lower resistivity diffusion barrier layer such as Co-W replacing TaN should meet the requirements for continuous Cu interconnect miniaturization. As the thickness of the diffusion layers decreases, the resistivity becomes increasingly more important to sustain the energy efficiency of the systems.

Choi et al. [28] have shown that for nanometric W films, both the Mayadas–Shatzkes and the Fuchs–Sondheimer equations fail to accurately predict the electrical resistivity at a specific thickness. Some authors have shown that for films of thickness closer to or greater than the material’s electron mean-free path, the dominant mechanism contributing to resistivity should be most affected by grain boundary scattering, as opposed to surface scattering [29,30,31,32,33]. In [33], the author affirms that the resistance of very thin lines should be proportional to λ × ρ_0_, where λ represents the element electron mean free path (mfp) and ρ_0_ its bulk resistivity. At room temperature, the electron mfp of Cu is 39.9 nm, whereas Co has an mfp value between 7.77–11.8 nm and W 15.5 nm [33]. As the Co and W electrons’ mean-free paths are well below the thickness of the barrier layer deployed, the predominant mechanism for diffuse electron scattering in this layer should be mostly independent of barrier layer thickness and rather predominantly a result of grain boundary scattering. In this way, a decrease in sheet resistance of films at the observed Cu thickness range of 40 nm should be attributed to grain growth, whilst rises in resistance should be associated with a decrease in Cu film thickness and potential increases in surface roughness [34]. This is supported by the fact that grain size tends to continuously grow as temperatures rise, whilst dewetting induces a decrease to film thickness, which in turn lead to higher resistivity values due to a considerable increase in electron–surface scattering. The minimum vacuum level of 1.20 × 10^−3^ Pa registered during annealing should minimize surface oxidation and its consequent increase in impurity scattering, which can be considerable for films of such reduced thickness as the ones studied [35]. However, for the 650 °C Cu/Ta sample, this did not prove to be the case. Both BSE SEM imaging and XRD results show that the purity level of the Cu film has been compromised by the presence of different chemical species, leading to an increase in the overall electrical resistivity even when copper dewetting did not seem to occur to the same extent as observed on the Cu/Co-W stack.

## 4. Conclusions

The findings in this work contribute to further the understanding of the performance of the Co-W system as an alternative material for diffusion barrier layer applications. The investigation results show that:

- From a Cu surface stability perspective, the Co-W system performed worse than the Ta film, with dewetting of the Cu layer in the 300–450 °C range;
- The Ta film revealed superior Cu dewetting resistance, with no discernible agglomeration at the surface after annealing for the studied temperature ranges;
- The Cu/Co-W system exhibited diffusion resistance up to 700 °C, whereas the Cu/Ta system failed in the 600–650 °C range, with Ta diffusion through the Cu layer;
- Co-W displayed structural continuity and integrity throughout the temperature range used;
- The sheet resistance of the Cu/Co-W was minimized after annealing at 450 °C, at 7.07 × 10^−6^ Ω/sq;
- The Cu/Ta system showed a minimum sheet resistance value of 6.03 × 10^−6^ Ω/sq after annealing at 450 °C. However, the need for the inclusion of a high resistance diffusion barrier layer such as TaN suggests an overall inferior electrical performance of the Cu/Ta/TaN system in comparison to the Cu/Co-W system.

## Figures and Tables

**Table 1:** The film thickness of the various samples analyzed bHAADF STEM imaging.

Film	Mean Thickness (nm)	Std. Deviation
Co-W (as-deposited)	26.9	1.8
Co-W (annealed 600 °C)	24.0	2.4
Cu (Co-W, as-deposited)	42.4	2.5
Cu (Co-W, 600 °C)	35.7	5.1
Ta (as-deposited)	23.0	1.2
Ta (annealed 600 °C)	17.8	2.3
Cu (Ta, as-deposited)	39.8	2.0
Cu (Ta, 600 °C)	41.0	2.6

**Table 2:** Electrical resistivity values for the calculated mean thickness values.

System	Electrical Resistivity (Ω∙cm)	
	25 °C	600 °C
Cu/Co-W	1.09 × 10^−5^	5.01 × 10^−6^
Cu/Ta	4.92 × 10^−6^	3.51 × 10^−6^

## Data Availability

The data presented in this study are available on request from the corresponding author.
